# Patterns of Skin Diseases in Qassim Province, Saudi Arabia

**DOI:** 10.7759/cureus.50125

**Published:** 2023-12-07

**Authors:** Yasmeen A Alfouzan, Lulwah S Alhumaidan, Maha Alsaif, Haitham Alsaif, Lamees Alharbi, Reem Almuhaymidi, Farees Y Almohaimeed, Fatimah A AlGhofaili

**Affiliations:** 1 College of Medicine, Qassim University, Buraydah, SAU; 2 College of Medicine, Unaizah College of Medicine and Medical Sciences, Qassim University, Buraydah, SAU; 3 Department of Dermatology, Qassim University, Buraydah, SAU

**Keywords:** saudi arabia, qassim, prevalence, alopecia, acne, dermatitis, pilosebaceous, dermatology

## Abstract

Objective

Dermatological complaints are one of the most common reasons to see a physician. Identifying the incidence and prevalence of different skin conditions is essential to improve health outcomes. Only a few studies regarding the pattern of skin diseases have been conducted in Saudi Arabia, especially in the Qassim region. This study aims to identify, evaluate, and compare the pattern of skin conditions in the Qassim region regarding age and sex, and to compare the results with previous studies.

Methods

A retrospective record-based study included all Saudi patients who attended the dermatology clinics at Qassim University Medical City, for 12 months, from 2021/08/26 to 2022/07/1. Data were collected from the electronic medical records.

Results

The study included 2775 Saudi patients comprising 1654 (59.6%) females and 1121 (40.4%%) males, with a male-to-female ratio of 1: 1.475. Around 75% of patients were between 15 and 34 years of age. The top most common diagnoses were pilosebaceous disorders (49.2%), with acne vulgaris being the predominant condition, followed by hair disorders (15.6%), dermatitis (9.2%), pigmentary disorders (7.2%), infections (3.9%), and papulosquamous disorders (3%). The prevalence of dermatological conditions was significantly higher in females than males for pilosebaceous disorders (P=.01) and hair disorders (P=.02).

Conclusion

There is a changing trend in the prevalence of skin disorders in the Qassim province of Saudi Arabia. Pilosebaceous disorders are being diagnosed more frequently compared to previous years and females visit dermatology clinics more than males. Due to its hospital-based setting, this study only gives a rough estimate of the pattern of skin diseases, and extensive epidemiological studies are needed to estimate the prevalence accurately.

## Introduction

Dermatological complaints are one of the most prevalent presentations in primary care offices and one of the most common reasons for general physicians’ referrals [1]. Skin conditions constitute a substantial proportion of primary care consultations worldwide, ranging from 24% to 37% [1,2]. In rural and far-flung areas, non-dermatologists, such as general physicians, would commonly hold the role of diagnosing and treating common skin disorders, with up to two-thirds of skin conditions treated by non-specialists [2]. Mild cases of common dermatological disorders like acne vulgaris, eczema, and skin infections can be managed in primary healthcare settings. However, in Qassim, they are often referred to dermatology clinics, posing a significant workload and delay in appointments [3].

Climate, ethnicity, hygiene, and educational level, among other factors, can influence the pattern of skin diseases in any community. These elements give each community its distinct pattern and explain the huge variation in reports from different parts of the world, and even within the same nation [4]. As a result, more pattern studies of skin disease in each geographic region are needed, as well as a greater emphasis on training non-dermatologists about certain common skin problems they are likely to encounter in their area [5].

Epidemiological population-based studies are the most effective method of investigating prevalence. Despite the significant burden of dermatological complaints in primary care, there is a notable lack of large-scale, population-based research focusing specifically on skin illnesses in Saudi Arabia [6]. Existing prevalence studies are predominantly hospital-based, thereby limiting their applicability to the actual socio-demographic strata of the country. Thus, this study aims to obtain contemporary data on the pattern of skin diseases based on age and sex differences in Qassim, to evaluate the change in pattern over time, and to compare the results with previous studies conducted in other regions of Saudi Arabia.

## Materials and methods

Study design

This retrospective study was conducted at Qassim University Medical City (QUMC) dermatology clinics. The study sample comprises all Saudi patients who attended the dermatology clinics during a period of one year, between 2021/08/26 and 2022/07/1.

Data collection

We collected data using the Electronic Health Record (EHR) database of QUMC, with approval from the Qassim University Medical Institutional Review Board (approval number: 21-23-04). A data extraction form was used to collect the data, which consists of the following: date of visit, age, gender, nationality, and the dermatological diagnosis made by a dermatology consultant or specialist. Data on all new and old patients during the study period were included. Non-Saudi patients, patients with non-dermatological diagnoses, and follow-up visits for the same complaint were excluded from the study. The dermatological diagnoses were categorized according to their class into one of the following: dermatitis, papulosquamous disorders, pilosebaceous disorders, pigmentary disorders, hair disorders, urticaria, keratinization disorders, viral, fungal, or bacterial infections. Any other complaint that did not fit in any of these classes, like scar conditions, was classified as others.

Statistical analysis

Data were entered into the Statistical Package for the Social Sciences (IBM SPSS Statistics for Windows, IBM Corp., Version 25.0, Armonk, NY). Univariate and multivariate analyses were used, in which the P-value and the 95% confidence intervals were evaluated from the analysis to show significance and correlation among variables. The P-value < 0.05 was taken as the fixed point for statistical significance.

## Results

Patient demographics based on age and gender

**Figure 1:**
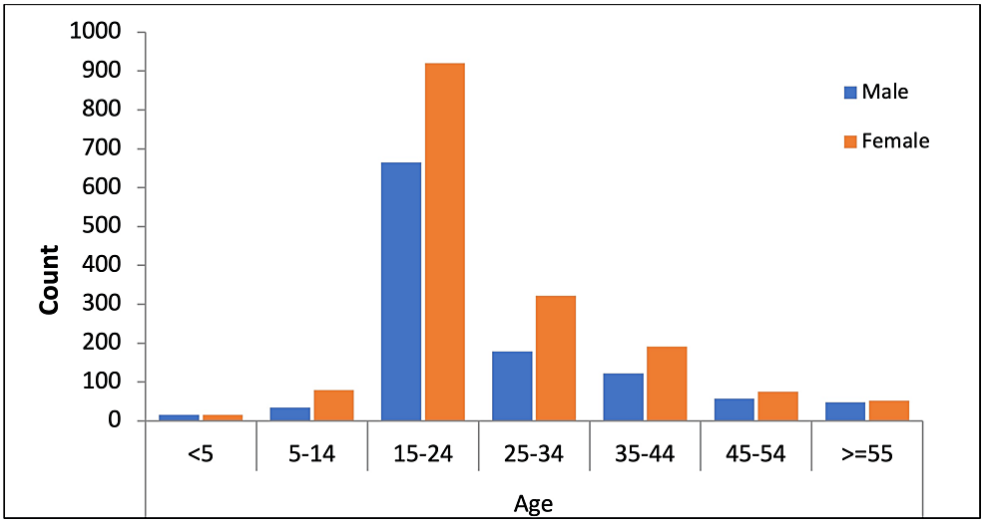
Distribution of patients by age and sex. The bar chart is plotted for age and gender depicting the male and female for different age ranges.

Out of 5813 appointments in the dermatology clinics during the study period, 2775 were included in this study. Excluded were non-Saudi patients (n = 1096), follow-up appointments for the same complaint (n = 1932), and diagnoses other than dermatological (n = 10). Male to female ratio was 1: 1.475, with females making up the majority (n=1654, 59.6%) compared to males (n=1121, 40.4%). Young adults (between 15 and 24 years of age) outnumbered all other age groups (n=1585, 57.1%), as shown in Figure 1. Females predominate in all age groups.

Different skin diseases among males and females

**Figure 2:**
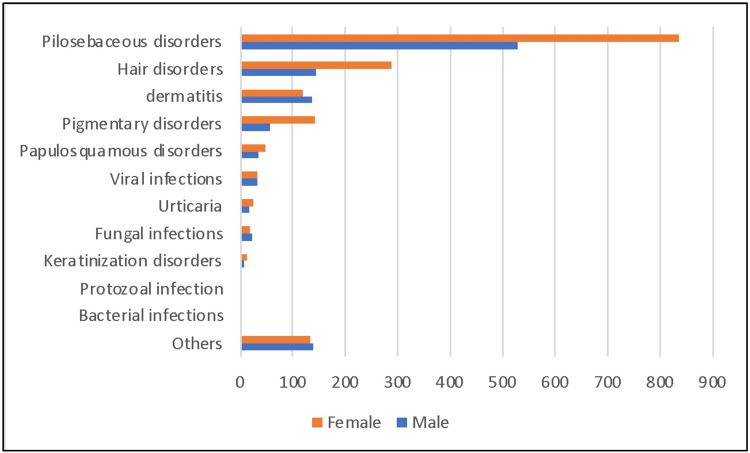
Overall ranking of skin disease and sex distribution.

The frequency of skin diseases by sex in order of ranking has been shown in Figure 2. It showed that females have a higher order of pilosebaceous disorder than males. The result is the same for all skin disorders except the one. Dermatitis has a higher frequency among males than females.

Skin diseases among the study population

The frequency of skin diseases seen in patients who visited the clinic is shown in Table 1. The most common skin disease was pilosebaceous disorders (n=1365, 49.2%), followed by hair disorders (n=434, 15.6%), and dermatitis (n=254, 9.2%). Of pilosebaceous disorders, acne vulgaris represents 97% of cases (n=1326). Telogen effluvium and androgenetic alopecia constitute 49% (n=212) and 27% (n=119) of hair disorders, respectively. The two most common dermatitis disorders were atopic dermatitis (n=79, 31%) and seborrheic dermatitis (n=63, 25%). Postinflammatory hyperpigmentation constitutes the majority of pigmentary disorders (n=92, 46%) and there were 37 cases of vitiligo (19%). Psoriasis was the most prevalent papulosquamous disorder (n=56, 67%). Viral infections were the most encountered infection in our study, predominantly warts (57 cases, 89%), with four cases of herpes simplex infection and two cases of chickenpox. This is followed by fungal infections. Dermatophytosis (n=29, 73%) and pityriasis versicolor (n=6, 15%) were the top forms of fungal infections. Only five cases of protozoal infections were diagnosed (four leishmaniasis and one pediculosis). Bacterial infections were the least common, with only one (0.01%) patient visiting the clinic.

**Table 1:** Skin diagnoses among the study population Frequency: number of cases Percent: percent of total cases

Skin diseases	Frequency	Percent
Pilosebaceous disorders	1365	49.2
Acne vulgaris	1326	47.8
Hair disorders	434	15.6
Telogen effluvium	212	7.6
Androgenetic alopecia	119	4.3
Eczema/dermatitis	254	9.2
Atopic dermatitis	79	2.8
Seborrheic dermatitis	63	2.3
Contact dermatitis	45	1.3
Pigmentary disorders	199	7.2
Postinflammatory hyperpigmentation	92	3.3
Vitiligo	37	1.3
Skin infections	110	3.9
Viral infections	64	2.3
Fungal infections	40	1.4
Protozoal infection	5	0.2
Bacterial infections	1	0.03
Papulosquamous disorders	83	3.0
Psoriasis	56	2.0
Lichen planus	7	0.3
Urticaria	41	1.5
Keratinization disorders	19	0.7
Others	270	9.7
Total	2775	100

Association of skin diseases with age and gender factors

The distribution of skin diseases among patients of different age groups and sexes has been shown in Table 2. Pilosebaceous disorders and hair disorders significantly correlate with sex, with females being more commonly affected (p=0.01 and p=0.02, respectively). There was no significant sex-specific variation in all other disorders. The most prevailing disease affecting most of the age groups was pilosebaceous disorder (n=1365, 49.2%), with the age group (15-24) being the most affected age group (p=0.001). No significant difference was observed among age groups in the prevalence of papulosquamous and pigmentary disorders.

**Table 2:** Multivariate analysis in association of skin diseases with age and gender factors Data represents the number of cases. P-value: significant value; CI: confidence interval

Specific diagnosis	Age									Gender			
	<5	5-14	15-24	25-34	35-44	45-54	>=55	P-value	CI	Male	Female	P-value	CI
Eczema/dermatitis	21	35	72	49	41	13	23	<0.001	4.22-4.43	136	118	0.06	3.36-3.74
Papulosquamous disorders	0	6	29	13	15	8	12	0.23	3.36-4.23	35	48	0.07	3.58-4.64
Pilosebaceous disorders	2	27	1007	218	74	32	5	0.001	1.23-1.34	529	836	0.01	1.36-2.34
Pigmentary disorders	1	9	76	38	43	23	9	0.21	2.33-2.56	56	143	0.06	2.01-2.36
Hair disorders	0	13	220	109	62	20	10	0.11	4.32-4.92	145	289	0.02	3.67-4.02
Urticaria	0	0	13	6	9	5	8	0.05	1.23-2.35	17	24	0.06	1.06-2.58
Viral infections	0	9	18	9	15	11	2	0.18	2.34-3.56	32	32	0.54	3.36-4.25
Fungal infections	1	1	13	8	7	2	8	0.25	3.87-4.27	22	18	0.21	3.58-4.20
Bacterial infections	0	0	0	1	0	0	0	0.72	3.56-4.26	1	0	0.34	3.50-4.21
Protozoal infection	0	1	4	0	0	0	0	0.34	1.12-2.69	3	2	0.03	1.56-2.98
Keratinization disorders	1	1	9	2	2	1	3	0.67	2.21-3.65	6	13	0.74	2.36-3.89
Others	5	11	124	48	45	17	20	0.55	3.11-3.29	138	132	0.27	3.01-3.59

## Discussion

The Qassim province is one of the 13 provinces of Saudi Arabia, landlocked at the heart of the country with a population of 1,370,727. It has a typical desert climate, known for its cool, rainy winters and hot, dry summers [7]. The study was conducted at QUMC, one of the largest hospitals in the region that serves all citizens and residents, primarily the university students, staff, and their families.

In our study, females comprised the major proportion of visitors to dermatology clinics compared to males. This is similar to some other Saudi studies conducted in Jeddah, Najran, Qunfudah, and Hail [8-11]. This may be because females are more conscious about their skin and body image and seek medical advice even for mild skin and hair problems or for prophylaxis. A study on the Saudi population concluded that females use more skincare products than males to enhance their skin appearance [12]. Nevertheless, a previous study conducted in the same center in 2009 showed the opposite results, where males comprised the major proportion of visitors (58.5%) [3]. It might be attributed to the global influence of social media focus on aesthetic image in recent years, as most of the female consultations are for pigmentary and hair problems.

In the presenting study, young adults visited dermatology centers more than the elderly or children. The bulk of our patients were between 15 and 25 years of age (57.1%), and about 70% were between 15 and 35 years old. This finding is similar to the previous study in Qassim, where patients from 15 to 34 years of age constitute 55% of the patients visiting the clinics [3]. This might be because people lose their concern about their skin when they become older or maybe during this period, acne and other common skin problems are less likely to occur. Also, it is worth mentioning that a large proportion of the patients visiting QUMC outpatient clinics are students of Qassim University.

There is a changing trend in the pattern of skin diseases in Qassim. The top most commonly diagnosed skin disorders in our study is pilosebaceous disorders, with almost half of the visits (49%), followed by hair disorder (15.6%), and eczema/dermatitis (9.2%). Compared to the previous study in Qassim, dermatitis was the top disorder (19.5%), followed by viral infections (16.6%), and pilosebaceous disorders (14.4%) [3]. According to a systematic review of studies from Saudi Arabia, the top five most commonly diagnosed skin disorders are diseases of skin appendages (24.8%), dermatitis (24%), skin infections (18.5%), pigmentary disorders (16.1%) and papulosquamous disorders (5.3%) [6]. This change in pilosebaceous disorder prevalence over time is similar to a study done in Hail in the period 2008-2014 for five years which found that 20% of patients attending dermatology clinics complain of acne vulgaris as compared to 12.43% of acne cases in the same hospital, King Khalid Hospital, in the period from 1995 to 1997 [11,13]. This is probably because most of our patients are adolescents and young adults, and the overall prevalence of acne vulgaris is increasing over time all around the world.

Dermatitis, also known as eczema, is the most common skin disorder in Saudi Arabia, with an estimated prevalence of 24% [4]. Studies conducted in Saudi Arabia showed constant but variable high prevalence in different regions, with a frequency of 21.4% in Jeddah [8], 48.2% in Qunfudah [9], 37% in Najran [10], 37% in Hail [11], 25.68% in Asir [14], and 19.6% as reported in Al-Khobar [15] and 19.5% in the previous study in Qassim [11]. Contact dermatitis is generally the most frequent type, while atopic dermatitis is observed more frequently in children [6]. In contrast, dermatitis was the third most commonly encountered complaint in dermatology clinics in our study comprising only 9.2% of cases, with atopic dermatitis followed by seborrheic dermatitis being the two most common types.

Interestingly, only 3.9% of patients in our study have skin infections. Viral, fungal, parasitic, and bacterial types constituted 2.3%, 1.4%, 0.2%, and 0.03% of the total consultations, respectively. Fungal infections were the top most common skin infections in Jeddah (10.9%), Al-Khobar (9.6%), and Qnfudah (6.4%) [8,15,9]. This might be because these are coastal cities with high humidity. In contrast, fungal infections represent 4.5% and 2% of total skin diagnoses in Riyadh and Hail, respectively [16,11], which has a similar low humid climate as in Qassim. In addition, most of the patients following up in QUMC are well-educated. In Cairo, infections were the most prevalent skin illness; similar findings have been recorded in other developing nations where poverty, a lack of knowledge, overcrowding, and inadequate hygiene are major factors [17,18].

In all dermatology consultations during the study period, only 12 cases of benign neoplasms were reported, all of which were cases of granulomas. In Saudi Arabia, skin cancers are generally uncommon, and melanoma is seldom seen [6]. Additionally, patients with connective tissue illness are directed to the rheumatology clinic at our hospitals, and patients with cutaneous neoplasms are referred to plastic surgery for diagnosis; as a consequence, very few of these patients visit the dermatology clinics.

This study has some limitations. External factors like the season of diagnosis, humidity, and temperature were not considered. In addition, our sample might not give an accurate representation of the Qassim population. This is because it was taken from the visitors of a university teaching hospital, where most of its clients are students and families of well-educated people.

## Conclusions

In conclusion, there is a changing trend in the prevalence of skin disorders in Qassim, Saudi Arabia, with acne vulgaris being diagnosed more frequently. Half of the patients complained of acne vulgaris, followed by hair disorders and dermatitis. Thus, training programs for primary healthcare physicians can reduce referrals and diagnose problems earlier. Further epidemiological studies are needed to accurately describe skin disease patterns in Qassim and other cities.
